# Antibody response to sand fly saliva is a marker of transmission intensity but not disease progression in dogs naturally infected with *Leishmania infantum*

**DOI:** 10.1186/s13071-017-2587-5

**Published:** 2018-01-04

**Authors:** Rupert J. Quinnell, Seyi Soremekun, Paul A. Bates, Matthew E. Rogers, Lourdes M. Garcez, Orin Courtenay

**Affiliations:** 1 School of Biology, Faculty of Biological Sciences, University of Leeds, Leeds, UK; 2 Zeeman Institute and School of Life Sciences, University of Warwick, Coventry, UK; 3 Faculty of Infectious Tropical Diseases, Department of Disease Control, London School of Hygiene and Tropical Medicine, London, UK; 4 Division of Biomedical and Life Sciences, Lancaster University, Lancaster, UK; 5 000 0004 0620 4442 Instituto Evandro Chagas, Belém, Pará Brazil; 6 Centro do Ciências Biológicas e da Saúde, Universidade do Estado do Pará, Belém, Pará Brazil

**Keywords:** Leishmaniasis, *Leishmania infantum*, Sand fly, Saliva, Antibody, Transmission, Exposure, Dogs, Brazil

## Abstract

**Background:**

Antibody responses to sand fly saliva have been suggested to be a useful marker of exposure to sand fly bites and *Leishmania* infection and a potential tool to monitor the effectiveness of entomological interventions. Exposure to sand fly bites before infection has also been suggested to modulate the severity of the infection. Here, we test these hypotheses by quantifying the anti-saliva IgG response in a cohort study of dogs exposed to natural infection with *Leishmania infantum* in Brazil.

**Methods:**

IgG responses to crude salivary antigens of the sand fly *Lutzomyia longipalpis* were measured by ELISA in longitudinal serum samples from 47 previously unexposed sentinel dogs and 11 initially uninfected resident dogs for up to 2 years. Antibody responses were compared to the intensity of transmission, assessed by variation in the incidence of infection between seasons and between dogs. Antibody responses before patent infection were then compared with the severity of infection, assessed using tissue parasite loads and clinical symptoms.

**Results:**

Previously unexposed dogs acquired anti-saliva antibody responses within 2 months, and the rate of acquisition increased with the intensity of seasonal transmission. Over the following 2 years, antibody responses varied with seasonal transmission and sand fly numbers, declining rapidly in periods of low transmission. Antibody responses varied greatly between dogs and correlated with the intensity of transmission experienced by individual dogs, measured by the number of days in the field before patent infection. After infection, anti-saliva antibody responses were positively correlated with anti-parasite antibody responses. However, there was no evidence that the degree of exposure to sand fly bites before infection affected the severity of the infection.

**Conclusions:**

Anti-saliva antibody responses are a marker of current transmission intensity in dogs exposed to natural infection with *Leishmania infantum,* but are not associated with the outcome of infection.

## Background

Haematophagous arthropod disease vectors inject saliva into hosts when blood-feeding that invokes anti-saliva immune responses. The detection of anti-saliva antibodies in host sera is a potential tool for monitoring changes in vector biting intensity brought about by seasonality, climatic conditions, or control interventions directed against the vector [1, 2]. The best current method for evaluating individual human exposure to vector bites is by human-landing catch counts using adult volunteers, but this method has ethical issues and does not provide measures for children who are often the high-risk group. Key to the useful application of an anti-saliva antibody immunoassay is having a precise understanding of the antibody kinetics in relation to changes in vector biting pressure. Such data are best quantified through a longitudinal study.

Phlebotomine sandflies are proven vectors for transmission of viruses (*Phlebovirus* and *Vesiculovirus*), bacteria (*Bartonella bacilliformis*), and protozoa (*Leishmania* spp.) that cause high morbidity, mortality and economic loss in humans, domestic animals, and livestock [3, 4]. Sand fly saliva contains some antigens, and both humans and other mammals produce a strong anti-saliva antibody response when naturally exposed to sand fly bites in endemic areas [5–10]. Experimental exposure of naïve hosts, including mice, dogs and humans, to sand fly bites shows that antibody responses to saliva are acquired rapidly, usually within a few weeks of exposure [11–16], and that the magnitude of the antibody response increases with the number of sand fly bites [11, 12, 15, 16]. The duration of antibody responses after the experimental challenge is more variable: no decline 24 weeks after exposure in BALB/c mice bitten by *Phlebotomus papatasi* [15], but a rapid decline in magnitude within a few weeks in dogs bitten by *P. perniciosus* and *Lu. longipalpis,* though responses remained positive in some dogs for up to 29 weeks [11, 12]. There have been fewer studies of the kinetics of antibody responses in naturally exposed hosts. Sentinel dogs and chickens develop antibody responses within a few months after their first exposure to sand fly bites [17, 18], and there is evidence of a decline in responses when humans and dogs cease to be exposed, though the magnitude of this decline is variable [8, 18]. Only one field study has compared anti-saliva antibody responses to an entomological measure of exposure; human anti-saliva antibody responses showed a positive but non-linear relationship with the number of female *Ph*. *argentipes* trapped inside houses in India and Nepal [8].

Exposure to sand fly bites also induces a strong cellular immune response to saliva [14, 19]. Studies of cutaneous leishmaniasis in rodent models have shown that exposure to sand fly bites, or immunization with salivary gland homogenate, prior to infection reduces the severity of subsequent experimental infection [20]. This effect is associated with a Th1 response to salivary antigens (reviewed by [19]). Pre-exposure to salivary gland proteins has also been associated with a lower severity of visceral leishmaniasis in the hamster model [21]. These results suggest that inclusion of salivary gland proteins in future vaccines could contribute to providing protection; partial protection against *L. major* infection has been shown recently in experimental macaques vaccinated with a *P. duboscqi* salivary protein [22]. However, it is less clear what the role is of sand fly induced host responses under natural conditions. Some cross-sectional field studies have shown that increased anti-saliva antibodies are correlated with the risk of infection [9, 23], and in some cases also the severity of infection [9, 23], but the interpretation of such cross-sectional studies is difficult. There have been no published longitudinal field studies that have compared the degree of pre-exposure to bites with the severity of natural *Leishmania infantum* infection.

Zoonotic visceral leishmaniasis (ZVL) is a fatal disease of humans caused by infection with *L. infantum*, for which the domestic dog is the principal reservoir [24]. Here we use data from a cohort of naturally infected dogs in Amazon Brazil to test whether the anti-saliva antibody response could be used as a marker of exposure to the sand fly vector *Lu. longipalpis*. The specific aims were to investigate (i) the rate of acquisition of responses in previously unexposed sentinel dogs; (ii) the rate of loss and re-acquisition of responses as a result of seasonal variation in transmission rates; and (iii) the relationship between the strength of the anti-saliva response and the force of infection experienced by each dog, measured by the time taken to become infected. We then test whether the degree of prior exposure to sand fly bites affects the severity of infection, by (iv) investigating the relationship between anti-saliva antibody responses and the severity of infection, assessed by parasite load and clinical symptoms.

## Methods

### Study site and study design

Serum samples were selected using archived material from a prospective cohort study carried out from April 1993 to July 1995 in the municipality of Salvaterra, Marajó Island, Pará State, Brazil (48°03'W, 00°46'S). The study design has been described previously [25, 26]. Briefly, 126 initially uninfected dogs were placed at intervals within households in the study site, and sampled approximately every 2 months (mean interval 67 days, range 58–80 days) during exposure to natural infection, for a maximum of 27 months. Details of the study design are provided in Table 1; most dogs were placed in the field in four initial cohorts, with the numbers in each cohort depending on the availability of dogs and households. Additional dogs were placed at later times when recipient households were available. Ninety-six dogs became infected during the study, and all dogs that were present for at least 8 sampling rounds became infected. Due to the limited amount of salivary antigen available, we could not test all samples, so used the inclusion criterion that dogs were sampled for at least 4 months (2 sampling rounds) after the date of infection. Samples from 61 of these 65 dogs were tested, in addition to samples from 2 other infected dogs. Unfortunately, the assay of one plate of samples did not succeed, which left 345 tested samples from 58 dogs, with an average of 6 samples tested per dog (range 3–11 samples). Eleven of the 58 dogs were initially uninfected resident dogs, born in the study area and so exposed to sand fly bites since birth. The remaining 47 were sentinel dogs taken to the study site from the nearby non-endemic city of Belém, and so had no previous exposure to sand fly bites. Time of patent infection in study dogs was defined using our previous results as the first time point of detection of *Leishmania* infection by any of the following methods: (i) detection of anti-*Leishmania* IgG by ELISA using crude leishmanial antigen (CLA), with antibody concentrations expressed as arbitrary units/ml relative to a positive control serum [25]; (ii) PCR on bone marrow biopsies using primers specific for kinetoplast DNA (kDNA) and ribosomal RNA [27]; (iii) quantitative kDNA PCR on bone marrow and ear skin biopsies, with results expressed as parasites/ml [28, 29]. All samples taken on or after the time of patent infection were classified as being from an infected dog. Negative control dogs comprised 6 unexposed, non-endemic UK dogs with no history of foreign travel that had attended two UK veterinary clinics during June to December 2007.

**Table 1:** Numbers of dogs that were enrolled in the study at each sampling point, and the numbers of dogs enrolled that were tested for anti-saliva antibody

Sampling round	Midpoint date	Day	No. of dogs enrolled	No. of tested dogs enrolled
1	11 April 1993	0	30	17
2	30 June 1993	80	21	14
3	28 August 1993	139	37	17
4	5 November 1993	208	16	4
5	13 January 1994	277	6	3
6	23 March 1994	346	2	1
7	30 May 1994	414	9	1
8	6 August 1994	482	0	0
9	12 October 1994	549	0	0
10	10 December 1994	608	5	1
11	19 February 1995	683	0	0
12	24 April 1995	746	0	0
13	6 July 1995	818	0	0
Total			126	58

### Sand fly salivary gland homogenate (SGH)

Salivary glands were dissected out from laboratory colony *Lu. longipalpis* sandflies (originally from Jacobina, Brazil), and stored in PBS in aliquots of 250 μl at a concentration of 200 pairs/ml. Glands were ruptured by 3 freeze-thaw cycles and the homogenate stored at -80 °C. The protein concentration of SGH was assayed using a standard BioRad protocol (BioRad Laboratories, Hercules, USA).

### Anti-*Lu. longipalpis* saliva ELISA

Ninety six-well ELISA plates (Linbro Scientific, Hamden, USA) were coated with salivary gland antigen at a concentration of 2.5 μg/ml in carbonate coating buffer (NaHCO_3_ 0.45 M, Na_2_CO_3_ 0.02 M, pH 9.6) at 100 μl per well, and incubated overnight at 4 °C. Plates were then washed 3 times with PBS-Tween (PBS pH 9.6 with 0.05% Tween-20). This was followed by blocking with 200 μl per well of 5% bovine serum albumin in PBS for 1 h at 37 °C. After 3 washes with PBS-Tween, 100 μl of sera (diluted 1:100 and 1:200 in PBS-Tween +0.5% BSA) was added and the plates incubated for 2 h at 37 °C. Two-fold serial dilutions (from 1:50–1:12,800) of serum, from a dog known to have a strongly positive reaction to saliva, were added to each plate as a standard curve. Following a further wash step, alkaline phosphatase-conjugated rabbit anti-canine IgG (Sigma-Aldrich, St. Louis, USA) was added at a dilution of 1:1000 in PBS-Tween-BSA to each well (100 μl/well) for 1 h at 37 °C. Following 3 further washes, 100 μl of p-nitrophenyl phosphate solution (Sigma-Aldrich) was added to each well and plates were left to develop at 37 °C for 20–30 min. Plates were stopped with 3 M NaOH and read at 405 nm. Negative control wells (no saliva antigen) were included for each sample dilution on the plate. Absorbance values of the negative controls were then subtracted from each corresponding sample. The resultant optical density (OD) values were then converted to units/ml using a log-logit transformed standard line. The positive control serum was assigned a value of 12,800 units/ml. All samples were above the lower detection limit of 100 units/ml; two samples above the higher detection limit were assigned the value of the higher detection limit (51,200 units/ml).

### Sample storage and quality control

Serum samples were collected during 1993–1995 and aliquoted at the time of collection. For long-term storage, samples were kept at -80 °C. CLA ELISA was carried out in 1996, and salivary gland ELISA in 2006. Samples had been briefly thawed several times by the time of salivary gland ELISA testing. Two years after the salivary gland ELISA analysis, a proportion of the total serum samples (*n* = 242, of which *n* = 131 were assayed for anti-saliva IgG) were re-tested by CLA ELISA in 2008 to confirm continued seroreactivity. A single sample showed strongly reduced reactivity; this sample was not part of the subsample tested for anti-saliva IgG in this study. The remaining samples showed a good agreement with the results of the initial CLA ELISA, with a strong and consistent positive correlation between antibody concentrations in 1996 and 2008 (*r*^2^ = 0.77). In 1996 and 2008 respectively, 162/241 (67%) samples and 159/241 (66%) samples were seropositive, with a high degree of concordance between years (kappa = 0.80) and no significant difference in sensitivity (McNemar’s test, *P* = 0.51).

### Measures of transmission intensity

The seasonal incidence of infection in study dogs was estimated between each sampling round from follow-up of uninfected dogs as described previously in [25]. The 11 fitted incidence estimates could be reduced to three without significantly reducing the fit [25], and here we use these three estimates to describe seasonal variation in incidence. Transmission intensity experienced by individual dogs was estimated as the incidence of infection of each dog, i.e. 1/(the number of days to patent infection for that dog), calculated from the time the dog was placed in the study area (Belém dogs) or the date of birth (resident dogs). Sand fly numbers are known to vary geographically across the study area [30]; geographical variation was modelled by using the village of residence as a variable. Sampled dogs came from 22 villages, with 1–7 sampled dogs per village. Data on seasonal variation of *Lu. longipalpis* numbers are available from a separate study that monitored sand fly numbers in 10 untreated chicken sheds in the same study area using CDC light traps at 8 time-points between October 1993 to June 1994 [31]. Precipitation data for 1993–1995 were obtained from the National Institute of Meteorology (MAARA), Belém, Brazil.

### Measures of infection severity

The severity of infection was assessed using (i) the magnitude of the anti-*Leishmania* IgG response, (ii) the parasite burden in bone marrow and ear biopsies assessed by qPCR as described earlier, and (iii) the clinical status assessed by a clinical score. For the clinical score, dogs were scored on a scale from 0 (absent) to 3 (intense) for six typical clinical signs of leishmaniasis (alopecia, dermatitis, chancres, conjunctivitis, onychogryphosis, and lymphadenopathy), and these scores summed. We used the maximum anti-*Leishmania* antibody level, bone marrow parasite burden and clinical score of each dog to classify infected dogs into three groups: severe, recovered and mild infection. Severe infection dogs were either (i) polysymptomatic (maximum clinical score of > 6) and with a high maximum antibody level and/or parasite load (defined as greater than the median of the maximum levels), or (ii) had high and increasing levels of both anti-parasite antibodies and parasites at their final sampling point, but were not polysymptomatic. Since clinical score typical increases after antibody and parasite levels, this latter group were expected to have become polysymptomatic. Recovered dogs had a maximum clinical score of > 6, but their final clinical score had reduced to < 3. Mild infections were defined as maximum clinical score < 7, and both anti-parasite antibody level and parasite load below the median.

### Statistical analysis

Anti-saliva antibody responses were analysed after log_10_-transformation, which normalised residuals and stabilised the variance. Statistical analyses of antibody responses against independent variables were conducted using linear models or linear mixed models. Mixed models with dog identity included as a random effect were used for all analyses when > 1 sample from each dog was included in the analysis; mixed models were fitted by maximum likelihood, and significance of explanatory variables tested by likelihood ratio tests (LRTs). To determine the effects of prior exposure to saliva on the severity of subsequent infection, anti-*Leishmania* antibody responses were analysed with linear mixed models after log_10_ transformation, while parasite burdens and clinical status (total clinical score) were analysed with negative binomial mixed models. Time (days) since the infection was controlled for by including linear and squared terms as covariates. For dogs where the response to saliva one round prior to infection was not assessed, this was estimated as the mean of the time points before and after this. The analysis was carried out in Stata 11.1, using the regress, xtmixed and xtnbreg routines (Stata Corporation, College Station, Texas, USA).

## Results

### Acquisition of anti-saliva antibody responses in sentinel dogs

**Fig. 1:**
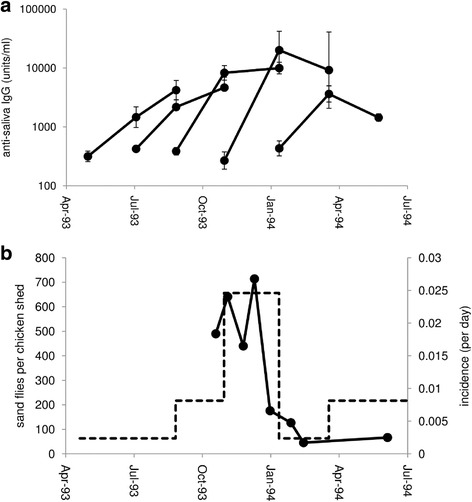
The initial acquisition of anti-sand fly saliva antibody responses in sentinel dogs. **a** The antibody response before exposure, 2 and 4 months after being placed in the field of each cohort of dogs (geometric mean ± SE). Sample sizes for each cohort are 11, 13, 15, 2 and three dogs; three further cohorts of a single dog each are not illustrated. **b** Seasonal changes in the estimated incidence of infection (dotted line) and numbers of *Lutzomyia longipalpis* sand flies caught in 10 chicken sheds (data from [31])

Anti-saliva antibody responses were measured in 47 sentinel dogs, and 11 resident dogs. Prior to exposure, i.e. before being placed in the field, sentinel dogs showed a low response (geometric mean 368 units/ml, SD range 211–642 units/ml), though somewhat higher than that of 6 non-endemic UK control dogs (geometric mean 183 units/ml, SD range 125–269 units/ml; *t*_(50)_ = 2.96, *P* = 0.005). Sampled sentinel dogs were placed into the field as 8 cohorts of 1–15 dogs at approximately 2-month intervals. Anti-salivary antibody responses rose rapidly in each cohort during the first 2 months in the field, with the mean increasing by 5 to 75-fold by the end of the first sampling period, an average of 65 days of natural exposure (paired t-test, *t*_(43)_ = 10.83, *P* < 0.0001; Fig. 1a). The magnitude of this initial rise within cohorts varied seasonally, with the highest rises observed in dogs placed in the field at the end of the calendar year (*F*_(7,37)_ = 4.47, *P* = 0.0011). This pattern reflected the seasonal variation in sand fly numbers and the incidence of infection, which peak at the end of the dry season in November-December and then decline rapidly (Fig. 1b). The initial rise in anti-saliva responses in each cohort of dogs was strongly associated with the log incidence of infection during that period (*F*_(1,43)_ = 30.3, *P* < 0.0001). After the initial rise, anti-saliva antibody levels did not show a consistent further increase during the next 65 days in the field, increasing in three cohorts and declining in two, in accordance with the seasonal pattern in sand fly numbers (Fig. 1a).

### Seasonal variation in anti-saliva antibody responses

**Fig. 2:**
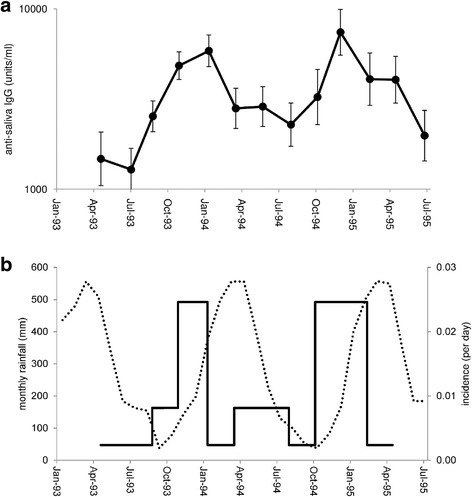
Seasonal variation in anti-sand fly saliva antibody responses. **a** Antibody responses (geometric mean ± SE). **b** Estimated incidence of infection (per day) in study dogs (solid line) and monthly rainfall in mm (dotted line) during the study period

Excluding the first (pre-exposure) sample from sentinel dogs, anti-saliva antibody responses were measured in 300 samples from 58 dogs, with an average of 5 samples per dog (range 3–10). The geometric mean anti-saliva antibody level (SD range) was 3607 (1053–12,362) units/ml. Follow-up of sentinel and resident dogs showed that after the initial acquisition of anti-saliva antibody, responses varied seasonally with the *L. infantum* transmission rate, declining sharply and significantly between January and March 1994, coincident with the decline in infection incidence associated with the start of the wet season (Fig. 2). This pattern was repeated the following year, with a significant increase from August to December 1994 at the end of the dry season, and then a decline from December 1994 to April 1995 during the following wet season (Fig. 2). Overall, anti-saliva antibody responses were significantly associated with the seasonal incidence rate at the time of the sample (LRT *χ*^2^ = 45.9, *df* = 1, *P* < 0.0001).

### Variation in anti-saliva antibody responses between dogs

**Fig. 3:**
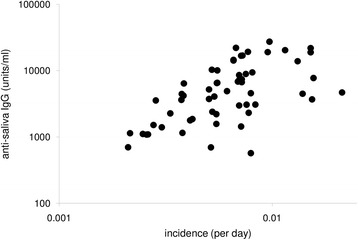
The relationship between the mean anti-saliva antibody responses of individual dogs and their incidence of infection. Incidence for each dog (*n* = 58) was assessed as the inverse of the number of days in the field to patent infection with *Leishmania infantum*

There was considerable variation between dogs in their mean antibody response to saliva, with a nearly 50-fold difference in mean levels (570 to 27,353 units/ml). This variation could reflect variation in exposure to sand fly bites or variation in host responsiveness. We used two surrogate measures of the biting rate per dog: the number of days each dog took to develop a patent infection (i.e. the force of infection experienced by each dog), and the village of residence (since sand fly numbers are known to vary geographically in the study area). Anti-saliva antibody responses were strongly associated with both the incidence of infection of the dog and with the village of residence, in addition to seasonal variations (sampling month) (Table 2). There was an increase in anti-saliva response with increasing transmission rate (Fig. 3) and mean anti-saliva responses varied up to 33-fold between villages. In contrast, anti-saliva responses were not associated with dog sex or origin (resident *vs* sentinel) (Table 2).

**Table 2:** Factors associated with the magnitude of the canine anti-saliva antibody response. Effect estimates are from an adjusted mixed model analysis with dog included as a random effect. Full data were available for 295 samples from 58 dogs

	Estimate (95% CL)	LRT *χ*^2^	*df*	*P*
Fixed effects				
Sampling round	–	119.8	12	< 0.0001
Incidence (1/days)^a^	1.392 (0.952–1.832)	28.73	1	< 0.0001
Village	–	46.94	21	0.001
Sex	0.020 (-0.153–0.193)	0.05	1	0.82
Origin	-0.093 (-0.339–0.153)	0.55	1	0.46
Random effect				
Dog	0.040 (0.024–0.066)^b^	60.44	1	< 0.0001
Residual	0.066 (0.055–0.078)^b^			

^a^log-transformed

^b^variance (95% CL)

### Relationship between anti-saliva responses and infection

Uninfected dogs with a high level of exposure to sandflies were more likely to become infected at their next sampling point: the odds of becoming infected by the next sampling round increased by 4.39-fold (95% CL: 1.79–10.74) with every unit increase in log anti-saliva IgG (logistic regression, LRT *χ*^2^ = 12.19, *P* = 0.0005). Infected dogs also had higher average anti-saliva responses than uninfected dogs (LRT *χ*^2^ = 17.86, *df* = 1, *P* < 0.0001), which reflects variation in transmission rate, and so this difference was not significant when controlling for seasonal effects (sampling month) (LRT *χ*^2^ = 0.76, *P* = 0.38). In infected dogs, there was a significant positive relationship between anti-saliva and anti-*Leishmania* IgG responses, but no relationship between contemporary anti-saliva IgG responses and *Leishmania* parasite burdens in tissue or clinical score (Table 3).

**Table 3:** Relationships between the severity of canine infection with *Leishmania infantum* and the anti-saliva antibody response, measured at the time of sampling, approximately 2 months before patent infection, and against transmission intensity (log incidence)

Severity measure	*n* (samples)	*n* (dogs)	LRT *χ*^2^	*P*
Anti-saliva IgG at time of sampling				
Anti-*Leishmania* IgG	206	58	7.11	0.0077
Parasites (bone marrow)	122	53	0.00	0.95
Parasites (ear skin)	80	40	0.03	0.86
Clinical score	195	58	0.00	0.97
Anti-saliva IgG prior to infection				
Anti-*Leishmania* IgG	374	58	2.79	0.095
Parasites (bone marrow)	214	55	0.00	0.97
Parasites (ear skin)	152	47	1.09	0.30
Clinical score	361	58	0.02	0.90
Transmission intensity (log incidence)				
Anti-*Leishmania* IgG	458	94	0.00	0.96
Parasites (bone marrow)	264	81	0.05	0.82
Parasites (ear skin)	184	63	1.51	0.22
Clinical score	439	92	1.04	0.31

**Fig. 4:**
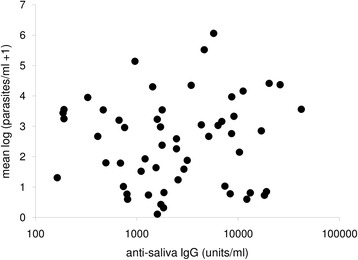
The relationship between the mean parasite load of individual dogs and their anti-saliva antibody responses 2 months before patent infection with *Leishmania infantum.* Parasite load was assessed as the mean log number of parasites in bone marrow biopsies of infected dogs (*n* = 55)

To examine possible effects of prior exposure to sand fly bites on the outcome of infection, we tested the relationship between the anti-saliva antibody responses measured 2 months before patent infection and the subsequent severity of the infection. No significant relationship was found, whether the severity of infection was assessed by the strength of the anti-*Leishmania* antibody response, by the parasite burden in bone marrow (Fig. 4) or in-ear skin biopsies, or by clinical score (Table 3). There was also no observed relationship between the transmission rate of each dog (measured by some days taken to become infected) and the severity of subsequent infection (Table 3).

**Fig. 5:**
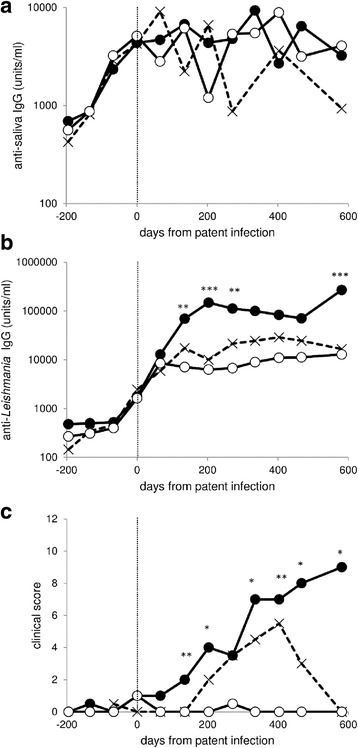
Variation in mean anti-saliva IgG responses (**a**), mean anti-*Leishmania* IgG responses (**b**) and median clinical score through time (**c**). Dogs were classified as severe infections (filled circles, *n* = 23), recovered (crosses, *n* = 6) and mild infections (open circles, *n* = 18). Estimated time of patent infection is indicated by the vertical dotted line. Asterisks indicate a significant difference between groups after sequential Bonferroni correction by ANOVA (IgG) or Kruskal-Wallis test (clinical score) *P < 0.05, **P < 0.01, ***P < 0.001

To further illustrate the lack of relationship between anti-saliva antibody levels and the outcome of infection, we classified infected dogs into severe (*n* = 23), recovered (*n* = 6) and mild infections (*n* = 18). The remaining 11 dogs could not be classified. These three groups had very different average antibody levels and clinical scores (Fig. 5), even though some dogs may have been misclassified, as dogs were not followed for the entire course of infection. However, there was no significant difference in anti-saliva antibody levels between the groups of dogs at any time-point, and levels before infection were very similar (Fig. 5).

## Discussion

We provide the first description of the kinetics of anti-sand fly saliva antibody responses in dogs exposed to natural transmission of *L. infantum* by *Lu. longipalpis*, and of the relationships between anti-saliva responses and the severity of the infection. The results suggest that the canine anti-saliva antibody response is a reliable marker of the magnitude of current exposure to sand fly bites and is thus a useful epidemiological tool for assessing community or individual exposure to infection, and the effects of interventions against sand fly biting rates within ZVL control programmes. Our results show that the strength of the anti-saliva antibody response before infection was positively associated with the risk of infection but, in contrast to experimental studies of murine leishmaniasis, there was no relationship between previous natural exposure to sand fly bites and the severity of *L. infantum* infection in this population.

Entomological measures of sand fly biting rate have some practical and ethical issues. The gold standard measure, the human landing catch, is time-consuming and ethically difficult, and cannot be performed on non-human hosts as human collectors are needed. Indirect measures using traps are logistically difficult. Antibodies to sand fly saliva have been suggested to be a useful marker for recent exposure to bites [5, 6]. A good surrogate measure of exposure would have a number of properties: the response should be rapidly acquired upon exposure; it should decline when exposure falls; there should be a positive, ideally linear, relationship between the magnitude of the response and the degree of exposure; and it should be easier to use than existing measures. Here, previously unexposed sentinel dogs acquired strong anti-saliva antibody responses within 2 months of exposure. Similarly, the rapid acquisition has been seen in experimentally exposed rodents, dogs and humans, with responses developing in two to 8 weeks [11–15]. The initial level of anti-salivary antibody responses in the sentinel dogs showed some responsiveness compared to UK dogs. This difference may reflect higher exposure to other biting arthropods such as mosquitos or fleas, or higher exposure to microbial antigens leading to generally upregulated antibody production. *Lu. longipalpis* has not been found in or around Belem, though some forest associated sand fly species may be found on the outskirts of the city.

To explore the loss of responsiveness as exposure decreases, we took advantage of the marked seasonal variation in sand fly abundance in the study area. Anti-saliva responses fell sharply by around 50% within 2 months at the onset of the wet season in the first months of 1994, coincident with the decline in both sand fly numbers and incidence of infection. A similar decline was seen the following year; though sand fly numbers were not monitored this year, the similar seasonal changes in incidence suggest that seasonal changes in sand fly numbers in each year were also similar. However, the decline in antibody response was less marked than the declines in sandflies and incidence, suggesting only a partial loss of titre. A similar pattern has been seen in experimentally exposed dogs, where the antibody response declined to about half the initial peak within a few weeks of the last exposure but remained at this lower level for at least 19 weeks [11, 12]. Anti-saliva antibody levels to natural exposure also showed a partial decline in both humans and dogs in the absence of bites, although the magnitude of this decline appeared to be less than in the current study [8, 18]. Therefore, where monitoring declines in incidence is important, such as during control programmes, using sentinel animals, e.g. chickens [17], placed in the field for only a short time-period may provide a more sensitive measure of change in exposure than monitoring resident animals with prolonged exposure. Experimental studies have shown that the relationship between the magnitude of the initial saliva antibody response and the number of sand fly bites is approximately linear in both mice and dogs [11, 12, 15]. The only previous field study of this relationship in naturally exposed hosts showed a positive, but non-linear, relationship between human antibody responses to *P. argentipes* and the numbers of sandflies captured within the household; though catches inside households may not be the best marker of exposure in that region [8, 32]. Here, we demonstrate a strong positive relationship between antibody responses and the incidence of *L. infantum* infection in dogs, both for the initial response and for responses in continually exposed endemic dogs, with a roughly linear relationship between the logarithms of antibody response and incidence. Although testing for anti-SGH antibodies was carried out on stored samples several years after collection, quality control showed that anti-leishmanial antibody levels in the stored samples remained high, with a strong correlation with results from the time of collection.

The major logistical issue for measuring anti-saliva responses is the limited availability of the antigen, which requires laborious dissection and preparation of sand fly SGH. In this respect, use of recombinant antigens would be advantageous; two recombinant *L. longipalpis* salivary antigens (rLJM11 and rLJM17) have been shown to have high sensitivity in humans when used in combination, and these antigens, and rLJL143, are also recognised by dogs [14, 33, 34]. In a recent study of a geographically different population of Brazilian dogs, we showed a close association (*r*^2^ = 0.77, *P* < 0.001) between IgG responses to SGH antigen and salivary recombinant proteins rLJM11 and rLJL143 (Bell & Courtenay, unpublished data). Similarly, canine responses to recombinant *P. perniciosus* antigens, and human responses to a recombinant *P. papatasi* antigen have been shown to correlate well with responses to SGH [18, 35–37]*.* Use of recombinant antigens should also improve the specificity of the assay [34], as cross-reactions between salivary antigens from related vector species may occur, such as *Lu. longipalpis* and *Lu. intermedia* [34], and *P. papatasi* and *P. argentipes* [8], which can complicate interpretation for areas where more than one sand fly species is common. However, in the current study area, lack of specificity was unlikely to be an issue, as *Lu. longipalpis* is the only abundant peridomestic species, comprising up to 99% of peridomestic captures [31, 38, 39]. Conversely, antigens from the whole saliva are more likely to be post-translationally modified and folded correctly and therefore active, with immunogenic proteins present in correct ratios. Care is needed in the selection of single proteins, or combinations, as they may elicit different or weak immune responses compared to SGH [34, 40, 41].

Anti-saliva responses varied greatly between individual dogs. Some of this variation may reflect differences in general antibody responsiveness between dogs, as the strength of response varies between dogs of a single breed experimentally exposed to a known number of bites [11]. However, the strong correlation with infection incidence shows that much of the variation is due to variation in exposure to sand fly bites. The observed variation between villages was consistent with the known variation in sand fly numbers across the study area: the villages with lowest antibody responses were in mostly open savannah grassland and riverine forest habitats, which have lower sand fly numbers [30], and also the peri-urban areas [42]. However, there was up to 20-fold variation in antibody response between dogs in the same village, which suggests a high variation in biting rate between dogs at small spatial scales, also consistent with high levels of variation in peridomestic sand fly numbers within villages [30]. These data indicate that a large sample size will be needed for precise measurement of community exposure. There was also a positive relationship between anti-saliva and anti-parasite antibody responses, previously shown in European dogs [18]. This is likely to reflect both the generalized up-regulation of antibody responses in visceral leishmaniasis and innate differences between dogs in their antibody responsiveness.

Exposure to sand fly bites, or immunization with SGH or defined antigens, has been shown to provide partial protection against subsequent infection with *L. major* and *L. amazonensis* in rodent models, with protection demonstrated by a reduction in pathology and/or parasite load (reviewed by [19, 20]), and also recently in non-human primates [22]. For *L. braziliensis* both protection and exacerbation of infection have been reported, depending on the species of sand fly saliva and type of antigen [9, 43]. Fewer studies have been performed using visceralizing parasites, but immunization with defined antigens from *Lu. longipalpis* saliva provided partial protection against infection with *L. infantum* in hamsters and enhanced protective immune responses in dogs [14, 21]. Confirmation of these effects in the field has proven difficult, in part because of the difficulty in distinguishing potential effects of exposure to sand fly saliva from those of transmission intensity [44]. In the present study, there was a strong positive association between the strength of anti-saliva responses before infection and the risk of being infected. Similar positive associations between anti-saliva responses and the presence or timing of infection have been seen in field studies of human cutaneous leishmaniasis and VL [5, 9, 10, 23, 44–46], though not in a study of European dogs [11]. However, such positive associations are expected, given variation in exposure to bites between hosts and between seasons; they do not imply that exposure to sand fly saliva increases the risk of infection, merely that hosts that are bitten more are more likely to become infected, since sand fly infection rates are low (typically < 2%) [47–49]. This positive association between anti-saliva responses and exposure to parasites complicates the identification of potential protective effects of saliva in the field, though there are suggestions of both protective and exacerbatory effects of saliva in human studies [9, 45].

To our knowledge, this study is the first field investigation to explicitly test the hypothesis that anti-saliva antibody responses before infection affect the severity of subsequent infection under endemic conditions. We found no relationship between prior exposure to sand fly bites, as measured by the strength of pre-infection anti-saliva antibody responses, and the severity of infection, assessed by parasite load or clinical score. We also found no relationship between infection outcome and the transmission rate. These results are based on measurements of a broad spectrum of immunological and parasitological infection outcomes following natural infection in outbred dogs and associated with carefully measured estimates of the temporal transmission intensity. There are several possible reasons why the protective effect of prior exposure to sand fly saliva seen in experimental infection was not seen in the field. One key factor is that, since natural sand fly infection rates are low, nearly all dogs will have been exposed to a large number of bites from uninfected sand flies, for an extended period, before they become infected. Chronic exposure to uninfected sand fly bites has been shown to remove the protective effect against experimental *L. major* infection [50]. It is also possible that more sophisticated measures of anti-saliva immunity may be necessary to demonstrate any effect since the protective effect in experimental infection has been shown to be due to cell-mediated, not humoral, immune responses to only certain sand fly antigens [14, 21, 41]. In endemic areas, hosts with a high biting rate will repeatedly be exposed to parasites once infected, which could also have a modulatory effect on the course of infection [51]. Moreover, there are some parasites released factors which accumulate inside the sand fly gut, such as proteophosphoglycans that form a gel (promastigote secretory gel) [52, 53] and protein-rich exosomes [54], that can be deposited during transmission and promote infection. Such factors may enable *Leishmania* infection even if exposure to sand fly saliva offers some protection. It is unlikely that selection bias in the current study would have weakened any relationship, as only dogs which died within 4 months of infection were excluded, which are unlikely to have died of leishmaniasis this rapidly after infection.

## Conclusions

The results confirm that anti-saliva antibody responses are a useful marker of exposure to sand fly bites in endemic areas, and could be used in epidemiological studies, to monitor changes in biting rate due to control measures [1], and to identify potential wild animal hosts [7, 55]. Development of recombinant antigen-based assays will be needed for widespread use, and the precise relationship between antibody levels and natural biting rate needs further study. Anti-saliva antibody responses were not associated with severity of subsequent infection in this study.

## Abbreviations

CDC: Centers for disease control and prevention
CLA: Crude leishmanial antigen
kDNA: Kinetoplast DNA
LRT: Likelihood ratio test
OD: Optical density
PCR: Polymerase chain reaction
SD: Standard deviation
SGH: Salivary gland homogenate
ZVL: Zoonotic visceral leishmaniasis
